# Promoting Prognostic Model Application: A Review Based on Gliomas

**DOI:** 10.1155/2021/7840007

**Published:** 2021-07-31

**Authors:** Xisong Liang, Zeyu Wang, Ziyu Dai, Hao Zhang, Quan Cheng, Zhixiong Liu

**Affiliations:** ^1^Department of Neurosurgery, Xiangya Hospital, Central South University, Changsha 410008, China; ^2^National Clinical Research Center for Geriatric Disorders, Xiangya Hospital, Central South University, Changsha 410008, China; ^3^Department of Clinical Pharmacology, Xiangya Hospital, Central South University, Changsha 410008, China

## Abstract

Malignant neoplasms are characterized by poor therapeutic efficacy, high recurrence rate, and extensive metastasis, leading to short survival. Previous methods for grouping prognostic risks are based on anatomic, clinical, and pathological features that exhibit lower distinguishing capability compared with genetic signatures. The update of sequencing techniques and machine learning promotes the genetic panels-based prognostic model development, especially the RNA-panel models. Gliomas harbor the most malignant features and the poorest survival among all tumors. Currently, numerous glioma prognostic models have been reported. We systematically reviewed all 138 machine-learning-based genetic models and proposed novel criteria in assessing their quality. Besides, the biological and clinical significance of some highly overlapped glioma markers in these models were discussed. This study screened out markers with strong prognostic potential and 27 models presenting high quality. Conclusively, we comprehensively reviewed 138 prognostic models combined with glioma genetic panels and presented novel criteria for the development and assessment of clinically important prognostic models. This will guide the genetic models in cancers from laboratory-based research studies to clinical applications and improve glioma patient prognostic management.

## 1. Introduction

Malignant tumors are characterized by therapeutic resistance, frequent recurrence, and distant metastasis, which cause difficulties in treating by either surgical resection or adjunctive therapies, leading to poor prognosis. For better clinical management, many prognostic models were proposed to analyze survival [1]. Previous models with unformulated predictors stratified patients into relative risk groups. However, they did not provide quantitative results or absolute risk stratification. Machine learning algorithms can identify critical patterns through big and complex data with high accuracy [2]. Common algorithms applied in cancer prediction include weighted gene coexpression network analysis (WGCNA), L1-penalized least absolute shrinkage selection operator (LASSO), Cox proportional hazards (PH) model [3], Neural Network [2], and Elastic regression [4]. Based on these machine learning algorithms, risk scores and further pictorial nomograms are constructed for addressing this issue [5, 6]. Risk score models are calculated with a spectrum of parameters in predicting clinical outcome risks. Samples are from local patient cases or online data. Establishing a model requires two main steps: development and validation [5]. First, predictors are selected and risks are calculated; thereafter, performance estimations are performed to assess the predictive quality. Second, the model is validated internally and externally (independent data set). Performance estimation is also performed [7].

**Figure 1:**
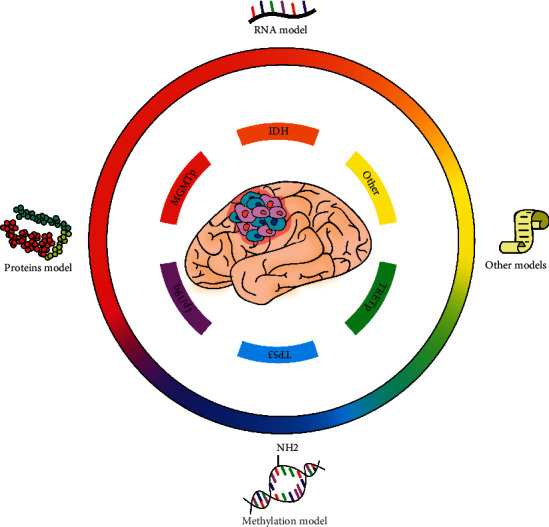
Glioma markers and genetic prediction models can stratify patient risks precisely and improve prognostic management. Glioma patient prognosis can be stratified and precisely predicted via prognostic markers and markers-based prediction models and, therefore, improved by adopting corresponding treatment strategies. Markers with prognostic values include IDH, MGMTp, 1p19q, TP53, TERTp, and many other molecules. Prediction models are based on RNA, protein, methylation, and other types of signatures. IDH, isocitrate dehydrogenase; MGMTp, O-6-methylguanine-DNA methyltransferase promoter; TP53, tumor protein p53; TERTp, telomerase reverse transcriptase promoter.

Gliomas are common intracranial tumors causing the highest mortality rates in all cancer types [8]. Routine treatments for gliomas include surgical excision, radiotherapy, and chemotherapy. The world health organization (WHO) grouped gliomas into four grades based on their histological features: WHO grades I, II, III, and IV [9]. Low-grade gliomas (LGGs) comprise WHO grades I and II, showing a relatively good prognosis. High-grade gliomas (HGGs) consist of WHO grades III and IV, manifesting worse survival outcomes [9]. Histological classification provides an understanding of glioma behavior. However, molecular groups differentiate prognostic groups more accurately [10] (Figure 1). The 2016 Central Nervous System WHO classification, therefore, provided molecular features including IDH-mutant/wildtype astrocytoma and glioblastoma, IDH-mutant/wildtype, and 1p/19q-codeleted/nonco-deleted oligodendroglioma, *H3K27M*-mutant/wildtype diffuse midline glioma, and RELA fusion-positive/negative ependymoma to precisely classify gliomas [11]. These and other markers such as *CDKN2A* and *EGFR* were combined to develop prognostic models for glioma patients [12].

**Figure 2:**
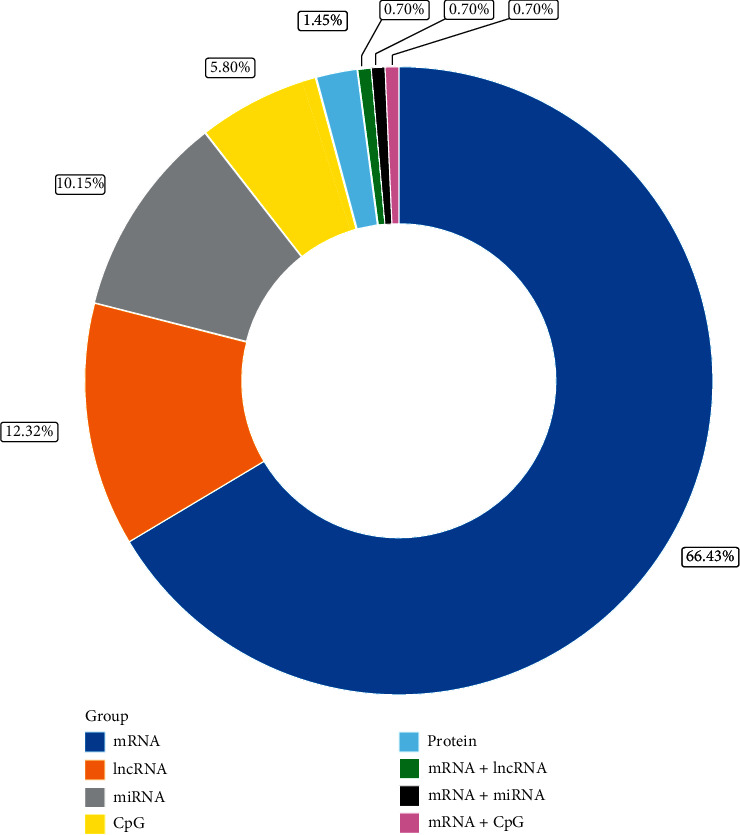
Proportion of published models based on signatures of different molecular types. The largest proportion of 94 mRNA models achieved 68% of all the 138 genetic prognostic models, followed by 17 lncRNA and 14 miRNA models, accounting for 12% and 10%, respectively. mRNA, message RNA; lncRNA, long noncoding RNA; miRNA, microRNA.

The poor prognosis of gliomas triggered the development of a clinically useful and effective model to assess survival risks for subsequent therapeutic strategies. This review systematically summarized and compared all 138 risk score models for gliomas with multivariable markers (Figure 2). It also presented the clinical significance of some frequently reported predictors, thus guiding the advanced prediction models for glioma. This will be conducive to translation from laboratory-based models to clinical available tools and clinical prognosis management improvement.

## 2. Rules for Evaluating Model Quality and Exclusion Criteria

**Figure 3:**
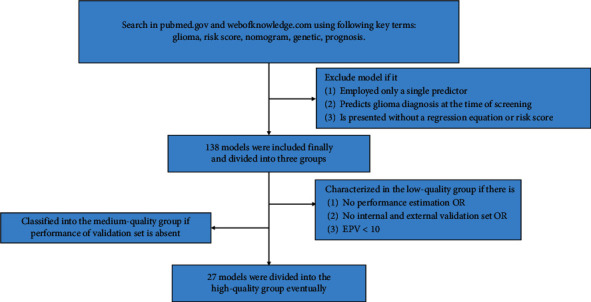
Procedure to screen and classify the genetic prediction models of glioma. The exclusion criteria and grouping rules are proposed firstly in this review, and details are described in Section 2. EPV, events per variable.

The TRIPOD statement standardized reports on the prediction models. It proposes a checklist of 22 items required in the development and validation process [13]. However, it does not judge model quality. Thus, we screened through the 138 models and proposed novel criteria that classified them into different quality groups (Figure 3); the criteria are listed in Table 1. After quality division, the high- and medium-quality models were discussed, and the low-quality group models were not presented (Table S1).

Performance estimation, validation, and EPV are vital factors to assess the model quality. Performance includes discrimination and calibration. Discrimination refers to the capability that differentiates patients from events that happen or not, which functions as the most essential quality results [14]. Calibration compares estimated event rates with observed rates [15]. Less efficacy arises when discrimination is confused with performance. Secondly, most studies lack calibration results [16]. For discrimination, we defined area under the curve (AUC), or c-index ≥ 0.80 as high accuracy, AUC ≥ 0.70 as acceptable, and AUC < 0.7 as low accuracies. The repeatability and transportability of the model should be verified through internal and external validation before clinical application [15]. Proper EPV requires 10 minimally determined by rule of thumb, but when many low-prevalence predictors are included, EPV should be up to 20 [17]; otherwise, it is considered an overfitting model.

Other aspects including variable number, missing value, outcome definition, reference genome, and annotation update also contribute to the model assessment. Excessive variables increase detective costs and decrease practicability. For most models contain less than 10 predictors and no certain conclusion have been presented, we defined less than 10 appropriate predictors. Missing data can lead to bias when not handled properly. Outcome definition is clearer and more informative with specific follow-up time than overall survival (OS). Reference genome and annotation updates can cause reversed individual risk prediction due to multiple gene expression diversity [18]. The inconsistent cutoff values between training and validation sets, screening method, and the threshold for predictors are complex questions and remain unresolved; they were not reviewed.

We excluded studies if (1) a single genetic predictor was employed with or without other types of predictors, (2) the model predicts glioma diagnosis at the time of screening, and (3) the model is inadequately presented without a regression equation or risk score.

## 3. RNA Models

### 3.1. mRNAs

Message RNA (mRNA) plays a critical role in central dogma that controls protein synthesis and decides cellular biology and behavior [19]. Many biological processes of mRNA in cancers, such as mRNA splicing, methylation, and interfering, can modulate the mRNA level and alter the cancer property; thereby, mRNA modulation has been a therapeutic target since early times. The levels of mRNAs in cancers, including gliomas, represent the expression of genes that are connected to the prognosis [11]. For glioma prognostic models, the largest proportion of models (78 models) are constructed based on mRNA sequencing data (Figure 2), of which 18 models are in the high-quality group.

#### 3.1.1. LGGs

Low-grade gliomas (LGGs) refer to WHO I, II gliomas [9]. Twenty-one mRNA models for LGGs have been reported. Among them, ten are in the high-quality group and nine are in the medium-quality group.

In the high-quality group, nine [20–28] and one [29] confer high and acceptable accuracy, respectively. Common advantages and additional luminous points in the high-quality group are shown. The corresponding AUC for multiple outcome events of 1, 3, and 5 years' survival in the 4 models [22–25] functions more powerful than many other models that predict merely OS. Two models were validated and assessed highly accurately both internally and externally [21, 22]. Su's model [25] is highly accurate, except when it predicted a 5-year survival training set (AUC was 0.711). For Zeng's model [24], despite its lower AUC for the external validation set, the nomogram performed better via c-index and calibration curve.

For the medium-quality group, five models exhibit high accuracy [30–34], three are acceptable [35–37], and one is lowly accurate [38]. Song et al. constructed a 21- [33] and a 20-gene model [34] both internally and externally, and the former was validated via four independent datasets. But the many numbers of predictors should be further streamlined. Hsu et al. [31] and Cheng et al. [30] values for AUC are extremely high (above 0.98 and 0.93, respectively). For deficiencies in this group, the seven models [30–32, 35–38] lack performance estimation in external or internal validation sets, and Ni et al. did not validate their 25-gene model [37].

#### 3.1.2. HGGs

High-grade gliomas (HGGs) comprise WHO III and Glioblastoma (GBM). A total of 44 HGGs models have been constructed but show a few satisfactory studies. The high-quality and medium-quality groups contain 7 and 11 models, respectively.

The high-quality models were designed for GBM [39–45]. 4-gene [44] and 3-gene [43] models associated with autophagy were both validated via two independent datasets. The two risk scores' predictive discrimination varies among survival rates in different years and datasets, while their nomograms stabilized the accuracy above 0.72, which integrated risk scores with other common factors. Nomograms' superior predictive accuracy to risk scores can also be observed in a study by Wang, the nomogram is highly accurate (from 0.77 to 0.85) compared with the AUC of risk score (from 0.67 to 0.79). Of note, nomogram performance decreased when estimated by c-index [39]. However, in a study by Zhu, the risk score outperformed the nomogram. AUC of risk score is 0.781 and 0.771 for 2 and 3 years of survival in the discovery cohort [41], while the c-indices of nomogram are also less than 0.70 [41]. Nomograms benefit from combining risk scores with other predictors, but whether their accuracy increases depends on the factors' quality. Moreover, the calibration curves were plotted and verified the reliability in the four studies [39, 41, 43, 44]. Additionally, 2329 samples from multiple cohorts in Zhu's study are the largest sample size in the currently existing models, improving its repeatability and transportability [41].

The medium-quality group is better since eight models are of high accuracy [46–53]. The AUC values in models developed by Zhang et al. [47], Hou et al. [52], and Chai et al. [48] are 0.93, 0.95, and 0.93, respectively. Two models achieved acceptable accuracy [54, 55], whereby Cheng's final model attained 0.81 when absorbing clinical features and 1p/19q status [55]. One model is lowly accurate [56]. This group is limited by a lack of performance estimation for the validation set except for Hou's model, but the weakness of Hou's model is the many predictors (14 genes) [52].

#### 3.1.3. Other Classified Gliomas Clusters

Three models and ten models are grouped into the high-quality and medium-quality groups from the total 28 models reviewed, respectively. The three high-quality models are highly accurate [57–59]. Particularly, Wang's model for diffuse glioma exhibits excellent discrimination with AUC from 0.874 to 0.950 [58]. But it was only internally validated.

Nine medium-quality models show high accuracy [60–68], whereby three models for diffuse glioma estimated accuracy level in training and validation sets despite the large size of predictors [64, 66, 67]. Notably, Sun's model has high accuracy when compared with all the signatures randomly derived from the screen method and outperformed the other three signatures in predicting drug sensitivity [66]. Additionally, it achieved higher accuracy when combined with age, grade, and another signature in the validation set for 3 and 5 years' survival [66]. Other 3 studies (two for all diffuse glioma [60, 65], and one for 1p/19q codeletion diffuse glioma) [62] together with a low-accuracy model for all glioma [69] in the medium-quality group are characterized by missing accuracy estimation in the validation set and excessive predictors.

Conclusively, most studies were designed for diffuse gliomas, and our criteria characterized 3 high-quality models. Besides, many models show high predictive accuracy when subjected to training. However, the absence of accuracy of validation sets failed to affirm the obtained discrimination results.

### 3.2. LncRNAs

Long noncoding RNAs (lncRNAs) are transcripts more than 200 nt in length. LncRNAs lack significant protein-coding capacity, but their regulatory functions are widely engaged from gene expression to protein translation. In gliomas, the lncRNAs function in stemness, drug resistance, blood–tumor barrier permeability, angiogenesis, and motility cancer phenotypes [70]. lncRNA is the second major hotspot in model research, after mRNA (Figure 2).

Seventeen lncRNA-signature models on different gliomas and one model on diffuse intrinsic pontine glioma have been reported. Three are high-quality models, and five are medium-quality models.

All three high-quality models exhibit high accuracy [71–73]. Except for the slightly decreased AUC values (0.722) when submitted to external validation based on Chen's study [73], the other two models show similar high accuracy in training, internal validation, and the entire set with AUC from 0.84 to 0.91 [71, 72].

The highest AUC value in the medium-quality group was obtained in Wang's model (0.942) for anaplastic glioma [74]. This is followed by Kiran's UVA8 model (8-lncRNA signature) [75] acceptably test for 5-year survival. The AUC values for the other two models were 0.68 and 0.70, respectively [76, 77]. In the five medium-quality models, they were externally validated, but three lacked internal validation [74, 77, 78]. Moreover, Kiran's study reported the UVA8 model and compared it with other predictors and models [75]. The UVA8 accuracy [75] is higher than other clinical features or IDH status. It outperformed the 5 published signatures in the training dataset by c-indices [75]. While the 6 models were designed for a diverse class of gliomas and different prognostic events and to validate various datasets, this positive result in Kiran's study was inevitably questionable due to incomparability.

Internal validation is absent in the 9 low-quality models (Table S1), which is vital to address the stability in selecting predictors and the quality of predictions before clinical application [79]. Cross-validation or bootstrapping methods should be employed to achieve complete internal validation.

### 3.3. miRNAs

MicroRNAs (miRNAs) are a class of noncoding RNA that binds to complementary target mRNAs. This results in mRNA translational inhibition or degradation. In gliomas, miRNAs are involved in various tumor-associated activities, including immune response, hypoxia, tumor plasticity, and resistance to therapy through multigene targets [80], indicating miRNA-based models as a promising strategy for glioma prognosis.

Fourteen studies on miRNA signatures, one on LGG [81], and others on HGG were reported. Three models were characterized in the high-quality group [81–83], whereas the other models were classified in the low-quality group.

The three high-quality models have respective advantages. The 5-miRNA model by Cheng et al. [83] adopted complete internal and external validations, and the AUC values increased from 0.649 and 0.756 to 0.847 and 0.909 after integrating age and chemotherapy, respectively, although only 19 samples were submitted to external validation. Chen's model [82] was internally and externally validated and achieved similar AUC for disease-free survival and OS in three datasets. Besides, Qian's study [81] established a nomogram that predicted a 1-, 2-, 3-, and 5-year survival rate, which is informative for prognostic management. However, its drawbacks include the absence of internal validation and low accuracy in the training set (c-index = 0.68).

## 4. Methylation Models

Cytosine-phosphate-guanine (CpG) islands are a cluster of CpG sites located at or near the transcription start regions of genes and gene promoters. CpG island methylation is the most common epigenetic type of cancer. The CpG island methylator phenotype that comprises several CpG islands is an interesting topic in cancer epigenetics [84]. The glioma CpG island methylator phenotype is associated with gliomas tumorigenesis and is an independent biomarker stratifying gliomas into epigenetic subtypes [85]. Currently, 8 models have been reported and none of them is a high-quality model; 3 of the models are classified into the medium-quality group [86–88].

The two medium-quality models have acceptable discriminations from 0.71 to 0.77 but were not internally validated [86, 87]. Yin's 6-CpG risk score [86] achieved a higher AUC value (0.734) for patients receiving all treatment integrated with the CpG island methylator phenotype. Moreover, higher AUC (0.771) was achieved with the *MGMT* status combination for those receiving radiation therapy/ temozolomide. Besides, the prediction accuracy rate of the 6-CpG signature (87%) was validated via the support vector machines model.

## 5. Other Multimolecular Models

Two protein-signature models based on reverse phase protein array were constructed. Stetson's 13-protein risk model [89] applied c-index to estimate the model's accuracy in both training and validating sets for GBM (0.63 and 0.60, respectively) and *IDH*-wildtype LGG (0.82 and 0.70, respectively), but the shortage is the low EPV (less than 10). Patil and Mahalingam developed another 4-protein model, but without external validation and performance estimation [90]. Both two models are of low quality.

Three mixed models of different classes of molecular signatures were presented for GBMs prognosis. The mixed model for mRNA and lncRNA is of high quality [91], and the other two are medium-quality models [92, 93]. The three models were fully validated both internally and externally. They were estimated using receiver operating characteristic curve or c-index; however, the validation set lacked estimation, and there was a low c-index value (0.68) in the training set from Etcheverry's study [92].

## 6. Biofunction and Clinical Significance of Frequently Reported Molecules

Molecular signatures reviewed consist of mutated genes, noncoding RNAs, and proteins. Currently, the star markers including *IDH*, *MGMT*, 1p/19q, *H3K27M*, *TERT*, and *ATRX* are known to exhibit significant prognostic value. The *IDH*-mutant with 1p/19q codeletion and *MGMT* promoter methylation are favorable prognostic factors. The *H3K27M*-mutant and ATRX alteration are associated with higher risk whereas *TERT* has a dichotomous prognostic effect [94]. Besides, other molecular signatures collected from 138 published models were reported to contribute to prognostic risk estimation. The prognostic value for most molecular biomarkers has not been validated. Therefore, we analyzed 138 models to select the most overlapping biomarkers (Table 2) with known evidence from researches to determine their biofunctions and potential prognostic values in gliomas. The predictors that presented repeatedly more than twice in 138 model studies are listed in Table 2. Predictors that overlapped less than three times were not reviewed.

### 6.1. *IGFBP2*

IGFBP2 regulates insulin-like growth factors' distribution and biofunction by interacting with the insulin-like growth factor system. High aberrant IGFBP2 expression was detected in HGG [95]. This played critical roles in glioma progression and was correlated with poor prognosis [95]. Besides, *IGFBP2* downregulation was reported specifically in IDH-mutant gliomas [96]. Besides, *EGFR* (epidermal growth factor receptor) and integrin*β* can integrate with *IGFBP2* to promote tumor progression [95]. The *IGFBP2* gene, therefore, presents prognostic value and functions as a potential immunotherapeutic target for GBM in the future clinical trials [95, 96].

### 6.2. *HDAC* Families and *CD44*

Histone deacetylase (HDAC) is a vast family of enzymes that mainly exert a repressive influence on transcription [97]. It blocks gene transcription by inhibiting histone acetylation and compacts the DNA/histone complex. In gliomas, *HDAC* functions to bridge the xCT-*CD44* complex with malignant glioma cells and various tumor zones [98]. Currently, *HDAC* inhibitors exhibit unfavorable therapeutic efficacy in glioma patients. Researchers have reported a more beneficial strategy that adopted *HDAC* inhibitors in combined therapy [99]. Moreover, many clinical trials (Table 3) are ongoing to test their application prospects in treating diffuse intrinsic pontine glioma. and HGG. Currently, *HDAC* inhibitors are characterized by *HDAC*s risk factors; however, their predictive ability has not been verified. In-depth explorations on their therapeutic efficacy and prognostic value are required.

*CD44* was identified in GBM and from brain metastases [100]. It is a biomarker of the mesenchymal GBM subtype with the most aggressive growth patterns [101]. Besides, GBM progression was inhibited by inhibiting *CD44* expression. This indicates the roles of *CD44* in the tumor process [102].

### 6.3. *MDK*

*MDK* is a heparin-binding growth factor encoding gene extensively studied for its multiple functions in various tissues. *MDK* contributes to numerous tumor-related activities in glioma, and its overexpression is associated with poor prognosis [103]. However, no clinical trial has been performed to explain its therapeutic potential.

### 6.4. *GPNMB*

*GPNMB* encodes the type 1 transmembrane protein expressed mainly on the surface of cancer cells [104]. *GPNMB* promotes tumor progression through immune-microenvironment plasticity. It also enhances Wnt/*β*-catenin signaling pathway activation and interaction with Na^+^/K^+^-ATPase subunits [105–107]. Also, abnormally high *GPNMB* expression has been reported to be associated with unfavorable survival outcomes in GBM [108]. These mechanisms justify that *GPNMB* is a risk factor for glioma patients.

### 6.5. *EGFR*

It has been reported that *EGFR* signaling pathways are activated in the majority of GBM cells [109]. EGFR gene aberration contains rearrangement, amplification, and mutation. The *EGFR* variant III (*EGFR*vIII) is a common mutation product in GBM [110]. The *EGFR*vIII contributes to many tumor biological features [111], indicating a poor outcome. Besides, wild-type *EGFR* associated with tumor cell invasion and angiogenesis has been demonstrated in several *in vivo* and *in vitro* experiments [112]. Thus, *EGFR* and *EGFR*vIII have been identified as popular therapeutic targets for treating malignant glioma patients. However, current treatments that target *EGFR* have failed in clinical trials including the small molecule drugs and biologic antibodies [112]. While some trials are still ongoing (Table 3).

### 6.6. *VEGFA*

*VEGFA*, also known as *VEGF*, is a growth factor that promotes tumor angiogenesis and vascular permeability and regulates immune cell and fibroblastoma and microenvironment formation [113]. In glioma, *VEGF* acts as a regulatory growth factor secreted by glioma stem cells to promote the tumor vasculature [114]. Anti-*VEGF*-A antibody has been applied as a novel antiangiogenic therapy for malignant gliomas, as with bevacizumab [114]. However, it has not achieved considerable progression in treating gliomas. A report by Eskilsson et al. [112] indicates that causes of failure may be attributed to enhanced invasive properties of tumor cells by different mechanisms, for example, RTK signaling. Many trials using bevacizumab with other strategies, for example, *EGFR* inhibitors, showed improved efficacy (Table 3); some trials are underway. Since several *VEGFA* studies on gliomas exist, it is expected that it will work as an accurate and effective predictor for glioma prognosis. This is despite its current poor performance in antiangiogenesis therapy.

### 6.7. miR-221/miR-222

miR-221/miR-222 are two closely related miRNAs located in the genome region of the X chromosome. They have a similar sequence, structure, and biofunction and upregulate expression in various human cancers including glioma. Knocking down miR-221/miR-222 expression blocks cell cycle transition, suppresses tumor cell growth, and increases sensitivity to radiotherapy [115]. Also, miR-221/miR-222 is associated with tumor cell apoptosis by targeting the apoptosis-related gene [116]. Recent studies revealed its connection with glioma histology and patient prognosis [117]. This showed that either miR-221 or miR-222 expression was associated with a higher WHO grade thereby indicating a poor prognosis.

### 6.8. Other Significant Predictors

Apart from the above-discussed vital parameters, other factors play significant roles in glioma malignant properties and prognosis assessment. These predictors include *MYC*, *KCNJ10*, *CHI3L1*, *STAT1*, and *FZD7. MYC* amplification has been identified in many classes of cancers. It acted as a qualified prognostic prediction biomarker [118]. *KCNJ10* encodes inwardly rectifying potassium channel Kir4.1 protein that is expressed exclusively in central nervous system glial cells. This establishes a hyperpolarized resting membrane potential and prevents glioma cell proliferation [119]. *CHI3L1* overexpression is associated with poor survival [120] causing tumor invasion, migration, angiogenesis, and resistance to temozolomide therapy [121]. The aberrant *STAT1* activation may cause oncogenesis [122]. Limited information on *FZD7* activity in gliomas exists; however, the other four predictors have attracted extensive research.

## 7. Discussion

For model variables, previous studies focused on pathological, anatomic, and clinical predictors, but recently genetic data were widely recommended for enhancing predictive ability [123, 124]. Formulated genetic predictors harbor an advantage in calculating absolute risks based on marker expression levels via sequencing analysis and coefficients, thus providing stronger information compared with relative risk tools. However, they are still not recommended for clinical application, due to limitations, such as the lack of gold markers, difficulties of data collection, the complexity of analysis, and low adherence to complete and transparent reporting [125]. Our review confirmed a similar problem in gliomas. We found that only 27 models (20%) are classified into the high-quality group (Table 4), 31 models (22%) are in the medium-quality group, and 83 models (58%) are classified in the low-quality groups; none can be clinically applied according to our criteria, which was urgently required to address the issue that no method for assessing the model quality exists currently [125].

Problems in models consist of methodological deficiencies and clinical confirmation absence. The former was mainly attributed to the low adherence to guidelines like the TRIPOD statement [13], leading to series of deficiencies like the lack of performance assessment, validation that was also observed in other cancers [123, 126, 127]. A good example of a prostate cancer model was constructed in transparent and clear details [128], and its constructing details were listed in a table, facilitating the check of model reliability for users and avoiding methodological negligence during the establishment process. And for validating, age-related risk score [129] was analyzed through 2953 cases from 10 datasets; the large sample size and high AUCs verified its robustness. For improving the model methodology, complete and transparent reporting should be strictly ensured. Besides, Zhang et al. highlighted that reference genome and annotation updates cause inconsistent gene expression levels, leading to discordant individual risk grouping. Using up-to-date reference genome, stable gene in each annotation release (with consistent length and overlap), and gene pairs is helpful [18]. For clinical application, while decision curve analysis [130] compares the net benefit of the models with traditional approaches, the best methods for testing clinical significance are prospect clinical trials [123, 125, 126]. Health economic impact evaluations should also be considered [131], but we found that no study has reported the cost of predictor detection. Economic cost and effectiveness of models were critical for the medical decision-maker, like cost-effectiveness ratio; they also contributed to the optimization of risk thresholds. However, since few studies have conducted these methods, we emphasized increasing awareness of clinical considerations when a high-quality model was established.

Conclusively, we comprehensively reviewed all 138 machine learning genetic models in gliomas and proposed novel criteria that will foster the development or assessment of clinically important models for not only neurosurgeons but also researchers in other cancers. Given the current situation, a lot of effort should be put to standardize the model quality through adherence to complete and transparent reporting and promote model generalization by conducting prospective clinical trials, and economic effectiveness should be the following issue. Despite the various difficulties, future genetic models will lead the prognostic management, and novel gene-pair-based models deserve development.

## Figures and Tables

**Table 1:** Criteria for model quality estimation.

Group	Items
High-quality group	EPV ≥10 AND
	Variable in final model <10 AND AUC/c-index estimated for both training and external or internal validation sets

Medium-quality group	EPV ≥10 AND
	Presence of external or internal validation set but only training set is estimated by receiver operating characteristic curve/c-index

Low-quality group	No performance estimation OR
	No internal and external validation set OR EPV <10

EPV, event per variable.

**Table 2:** Highly overlapping genes and miRNAs.

Overlapping times	Molecule numbers	Genes	miRNAs
6	1	IGFBP2	—
5	3	HDAC, STAT1	miR-222
4	6	GPNMB, VEGFA, EFEMBP2, ISG20, FZD7, EGFR,	—
3	23	MDK, CHI3L1, LGALS3, IFI44, OSMR, MYC, TOP2A, CD44, LDHA, BMP2, KI67, BUB1, LAMB1, MAP2K3, KCNB1, KCNJ10, IRF1, ASF1A, SOCS3, KCNAB1	miR-148a, miR-15b, miR-145, miR-20a

**Table 3:** Completed and active trials primarily completed in 5 years of HDAC, VEGF, and EGFR related therapy.

Gene	Agent name	Glioma type	Clinical trials	Development phase	Status
HDAC	Vorinostat	DIPG	NCT01189266	Phase I/II	Active
		DIPG	NCT02420613	Phase I	Active
		HGG	NCT01236560	Phase II/III	Completed
		HGG, recurrent GBM	NCT01266031	Phase I/II	Completed
	Belinostat	GBM	NCT02137759	Phase II	Active

VEGF	Bevacizumab	Anaplastic glioma, recurrent GBM	NCT01836536	Unknown	Completed
		Recurrent ependymoma, WHO II, III glioma	NCT00381797	Phase II	Completed
		GBM	NCT01091792	Early phase I	Completed
		HGG, recurrent GBM	NCT01266031	Phase I/ II	Completed
		HGG	NCT01236560	Phase II/III	Completed
		Recurrent GBM	NCT01648348	Phase I/II	Completed
		GBM	NCT01498328	Phase II	Completed
		GBM, oligodendroglioma	NCT01609790	Phase II	Active
		Recurrent GBM	NCT02142803	Phase I	Active
		Recurrent GBM	NCT02974621	Phase II	Active

EGFR	Erlotinib	HGG	NCT01257594	Phase I	Completed
	Lapatinib	Recurrent HGG	NCT02101905	Phase I	Active
	Unknown^†^	GBM	NCT01454596	Phase I/II	Completed
	AMG 595	Recurrent GBM, AA	NCT01475006	Phase I	Completed
	Sym004	Recurrent GBM	NCT02540161	Phase II	Completed
	Depatux-M	GBM	NCT02343406	Phase II	Completed
		GBM	NCT02573324	Phase II/III	Active
		GBM	NCT03419403	Phase III	Terminated
		GBM	NCT01800695	Phase I	Completed
	Dacomitinib	Recurrent GBM	NCT01520870	Phase II	Completed
		GBM	NCT01112527	Phase II	Completed
	Tesevatinib	GBM	NCT02844439	Phase II	Completed
	Afatinib	GBM	NCT00977431	Phase I	Completed
	Cetuximab	GBM, anaplastic astrocytoma	NCT01238237	Phase I	Completed
	Rindopepimut	GBM	NCT01498328	Phase II	Completed
		GBM	NCT01480479	Phase III	Completed

DIPG, diffuse intrinsic pontine glioma; HGG, high-grade glioma; GBM, glioblastoma; AA, anaplastic astrocytoma. ^†^Epidermal growth factor receptor (EGFRv) III; chimeric antigen receptor (CAR); transduced peripheral blood lymphocytes (PBL).

**Table 4:** Characteristics of 27 high-quality models.

Authors	Glioma type	Parameters	Sample size^a^	Validation^b^	Performance estimation^c^	Accuracy^d^	Reference
Liu et al.	LGG	2 mRNAs	115/-/41	E	T, E	Low (0.735)	[29]
Xiao et al.	LGG	3 mRNAs	456/-/159	I, E	T, E	High (0.908, 0.878, 0.827)^e^	[23]
Chen et al.	LGG	3 mRNAs	164/-/599	E	T, E	High (0.869)	[28]
Zeng et al.	LGG	4 mRNAs	172/-/451	E	T, E	High (0.845, 0.890, 0.912)^e^	[24]
Zhang et al.	LGG	4 mRNAs	534/-/325	E	T, E	High (0.858, 0.853, 0.837)^e^	[27]
Liu et al.	LGG	5 mRNAs	524/-/169	I, E	T, E	High (0.887)	[26]
Li et al.	LGG	4 mRNAs + 6mRNAs	516/-/426	E	T, E	High (0.84)	[20]
Zhang et al.	LGG	6 mRNAs	304/128/353	I, E	T, I, E	High (0.914)	[21]
Zhang et al.	LGG	7 mRNAs	297/124/353	I, E	T, I, E	High and acceptable (0.899, 0.875, 0.778)^e^	[22]
Su et al.	LGG	8 mRNAs	511/-/172	E	T, E	High and acceptable (0.882, 0.831, 0.711)^e^	[25]
Qian et al.	LGG	4 miRNAs	100/-/420	E	T, E	Low (0.680)	[81]
Wang et al.	GBM	3 mRNAs	155/-/216	E	T, E	High (0.832)^f^	[43]
Wang et al.	GBM	4 mRNAs	241/160/-	I	T, I	High and acceptable (0.756, 0.821, 0.885)^ef^	[44]
Wang et al.	GBM	5 mRNAs	364/155/-	I	T, I	Acceptable (0.729)^f^	[39]
Zhao et al.	GBM	6 mRNAs	152/-/138	E	T, E	High (0.908)	[40]
Zhu et al.	GBM	5 mRNAs	306/325/1957	I, E	T, I, E	Acceptable (0.704)^f^	[41]
Zuo et al.	GBM	6 mRNAs	137/-/158	E	T, E	Acceptable (1-year 0.699,2-year 0.779)	[42]
Gong et al.	GBM	8 mRNAs	151/-/138	E	T, E	High (0.977)	[45]
Zhou et al.	GBM	6 lncRNAs	200/219/-	I	T, I	High (0.902)	[72]
Chen et al.	GBM	4 lncRNAs	240/-/80	E	T, E	High (0.843)	[73]
Chen et al.	GBM	7 miRNAs	89/102/109	I, E	T, I, E	Low (0.690)	[82]
Cheng et al.	GBM	5 miRNAs	75/75/19	E	T, E	High (0.847)^f^	[83]
Gao et al.	GBM	6 mRNAs + 5 lncRNAs	76/77/80	I, E	T, E	Acceptable (0.780)	[91]
Peng et al.	Diffuse glioma	5 mRNAs	641/-/319	E	T, E	High (3-year 0.895, 5-year 0.864)	[59]
Wu et al.	Glioma	9 mRNAs	550/-/309	E	T, E	High (0.860)	[57]
Wang et al.	Glioma	5 mRNAs	420/178/-	I	T, I	High (0.917, 0.950, 0.881)^e^	[58]
Hu et al.	Glioma	5 lncRNAs	70/70/-	I	T, I	High (0.910)	[71]

^a^Sample size of training set/internal validation set/external validation set. ^b^I and E represent the presence of internal and external validation, respectively. ^c^T, I, and E represent the presence of performance estimation in training, internal validation, and external validation sets, respectively. ^d^Models were classified into high, acceptable, and low accuracy according to AUC or c-index as we present in rules for judging model quality and excision criteria part, and accuracy in only the training set is shown. ^e^Three AUC values for prediction of 1-, 3-, and 5-year survival, respectively. ^f^If higher accuracy was achieved by nomogram or integrated model, the highest accuracy result was exhibited instead of the result of the original risk score.

## Data Availability

No data were used to support this study.

## Abbreviations

AUC: Area under the curve
*EGFR*: Epidermal growth factor receptor
EPV: Events per variable
GBM: Glioblastoma multiforme
HGG: High-grade glioma
LGG: Low-grade glioma
lncRNA: Long noncoding RNA
miRNA: Micro RNA
OS: Overall survival
WHO: World Health Organization.

## Supplementary Materials

Supplementary Materials Table S1: characteristics of 73 models in the low-quality group.
