# Supplementary material for: Promoting Prognostic Model Application: A Review Based on Gliomas
Source: J Oncol. 2021 Jul 31;2021:7840007. doi: 10.1155/2021/7840007 (PMC8356003; doi:10.1155/2021/7840007)
Supplement: Supplementary Materials — Table S1: characteristics of 73 models in the low-quality group. [file 7840007.f1.docx]

Supplementary Table S1 **Characteristics of 73 models in the low-quality group.**

| Authors | Glioma type | Parameters | Main disadvantages^†^ | Advantages^‡^ | Reference | |
| --- | --- | --- | --- | --- | --- | --- |
| Liang et al | GBM | mRNA | A, P | A novel REST score | | [[132](#_ENREF_132)] |
| Colman et al | GBM | mRNA | A | Complete validation | | [[133](#_ENREF_133)] |
| Fatai et al | GBM | mRNA | A, P | Complete validation | | [[134](#_ENREF_134)] |
| Luedi et al | GBM | mRNA | A, P, V |  | | [[135](#_ENREF_135)] |
| Zhao et al | GBM | mRNA | A, P, |  | | [[136](#_ENREF_136)] |
| Cao et al | GBM | mRNA | A |  | | [[137](#_ENREF_137)] |
| Arimappamagan et al | GBM | mRNA | P | Complete validation | | [[138](#_ENREF_138)] |
| Chen et al | GBM | mRNA | A | Complete validation, multiple independent validation | | [[139](#_ENREF_139)] |
| Scott et al | GBM | mRNA | A, E, V |  | | [[140](#_ENREF_140)] |
| Hu et al | GBM | mRNA | A, P, |  | | [[141](#_ENREF_141)] |
| Xu et al | GBM | mRNA | V | High AUC | | [[142](#_ENREF_142)] |
| Vauleon et al | GBM | mRNA | A | Multiple independent validation | | [[143](#_ENREF_143)] |
| Huang et al | GBM | mRNA | A, P |  | | [[144](#_ENREF_144)] |
| Ahmed et al | GBM | mRNA | A, V |  | | [[145](#_ENREF_145)] |
| Yang et al | GBM | mRNA | V |  | | [[146](#_ENREF_146)] |
| Sun et al | Diffuse glioma | mRNA | A, P |  | | [[147](#_ENREF_147)] |
| Chang et al | GBM, LGG | mRNA | P, V |  | | [[148](#_ENREF_148)] |
| Wang et al | GBM | mRNA | A, P | Complete validation | | [[149](#_ENREF_149)] |
| Cheng et al | GBM | mRNA | A |  | | [[150](#_ENREF_150)] |
| Celiku et al | GBM, grade Ⅱ | mRNA | A, E, P |  | | [[151](#_ENREF_151)] |
| Zhou et al | Glioma | mRNA | A, P |  | | [[152](#_ENREF_152)] |
| Bao et al | Diffuse glioma | mRNA | A, E | Multiple independent validation | | [[153](#_ENREF_153)] |
| Wang et al | All gliomas | mRNA | A, P | Multiple independent validation | | [[154](#_ENREF_154)] |
| Lu et al | Diffuse glioma | mRNA | A, P | Nomogram construction | | [[155](#_ENREF_155)] |
| Zhang et al | All gliomas | mRNA | V |  | | [[156](#_ENREF_156)] |
| Pitroda et al | Glioma | mRNA | A |  | | [[157](#_ENREF_157)] |
| Wu et al | All gliomas | mRNA | A |  | | [[158](#_ENREF_158)] |
| Bhargava et al | GBM | mRNA | A, E |  | | [[159](#_ENREF_159)] |
| Wang et al | Diffuse glioma | mRNA | A |  | | [[160](#_ENREF_160)] |
| Zhang et al | HGG | mRNA | A |  | | [[161](#_ENREF_161)] |
| Zhou et al | Astrocytic, Oligodendroglial | mRNA | E, P | Complete validation | | [[162](#_ENREF_162)] |
| Wani et al | Infratentorial ependymoma | mRNA | A |  | | [[163](#_ENREF_163)] |
| Qian et al | LGG | mRNA | A, P | Multiple independent validaiton | | [[164](#_ENREF_164)] |
| Ni et al | LGG | mRNA | E, P |  | | [[37](#_ENREF_37)] |
| Trong et al | IDH-mutant glioma | mRNA | A |  | | [[165](#_ENREF_165)] |
| Glinsky et al | Medulloblastoma, glioma | mRNA | A, P, V |  | | [[166](#_ENREF_166)] |
| Hu et al | Diffuse glioma | mRNA | A, E |  | | [[167](#_ENREF_167)] |
| Li et al | Glioma | mRNA | A |  | | [[168](#_ENREF_168)] |
| Chen et al | GBM | mRNA | A |  | | [[169](#_ENREF_169)] |
| Wang et al | GBM | mRNA | A |  | | [[170](#_ENREF_170)] |
| Kawaguchi et al | GBM | mRNA | A, E, P |  | | [[171](#_ENREF_171)] |
| Wang et al | GBM | mRNA | A |  | | [[172](#_ENREF_172)] |
| Cai et al | GBM | mRNA | A |  | | [[173](#_ENREF_173)] |
| Yan et al | Glioma | mRNA | P |  | | [[174](#_ENREF_174)] |
| Chen et al | Recurrent GBM | mRNA | P | Complete accuracy assessment | | [[175](#_ENREF_175)] |
| Yan et al | GBM, grade Ⅲ | miRNA | A |  | | [[176](#_ENREF_176)] |
| Srinivasan et al | GBM | miRNA | A |  | | [[177](#_ENREF_177)] |
| Zhang et al | GBM | miRNA | E |  | | [[178](#_ENREF_178)] |
| Sathipati et al | GBM | miRNA | A, P |  | | [[179](#_ENREF_179)] |
| Sana et al | GBM | miRNA | A, E |  | | [[180](#_ENREF_180)] |
| Li et al | GBM | miRNA | A |  | | [[181](#_ENREF_181)] |
| Hayes et al | GBM | miRNA | A | Complete validation | | [[182](#_ENREF_182)] |
| Niyazi et al | GBM | miRNA | A, E |  | | [[183](#_ENREF_183)] |
| Hermansen et al | GBM | miRNA | E |  | | [[184](#_ENREF_184)] |
| Hua et al | GBM | miRNA | A, E |  | | [[185](#_ENREF_185)] |
| Sana et al | GBM | miRNA | A |  | | [[186](#_ENREF_186)] |
| Wang et al | Grade Ⅲ | lncRNA | A, E |  | | [[187](#_ENREF_187)] |
| Liu et al | DIPG | lncRNA | E, V |  | | [[188](#_ENREF_188)] |
| Reon et al | LGG | lncRNA | A, P |  | | [[189](#_ENREF_189)] |
| Liang et al | GBM | lncRNA | A |  | | [[3](#_ENREF_3)] |
| Paul et al | GBM | lncRNA | A, V |  | | [[190](#_ENREF_190)] |
| Xian et al | Glioma | lncRNA | A |  | | [[191](#_ENREF_191)] |
| Luan et al | All gliomas | lncRNA | A |  | | [[192](#_ENREF_192)] |
| Lin et al | Diffuse glioma | lncRNA | A, P |  | | [[193](#_ENREF_193)] |
| Cao et al | GBM | lncRNA | A |  | | [[194](#_ENREF_194)] |
| Bady et al | IDH-mutant LGG | CpG | A | Multiple independent validation | | [[195](#_ENREF_195)] |
| Shukla et al | GBM | CpG | E | Complete validation | | [[88](#_ENREF_88)] |
| Yin et al | GBM | CpG | A | Complete validation | | [[196](#_ENREF_196)] |
| Liao et al | Methylated LGG and GBM | CpG | A, P | Multiple independent validation | | [[197](#_ENREF_197)] |
| Chai et al | MGMTp Unmethylated GBM | CpG | A | Complete validation | | [[198](#_ENREF_198)] |
| Kang et al | non‐G‐CIMP GBM | CpG | A, P, V |  | | [[199](#_ENREF_199)] |
| Stetson et al | GBM | protein | P | Complete validation, accuracy assessment | | [[89](#_ENREF_89)] |
| Patil et al | LGG | protein | A |  | | [[90](#_ENREF_90)] |

^†^ A, lack of performance assessment; E, low events per variable; P, excessive predictors; V, lack of any validation;

^‡^ Complete validation, model was validated both internally and externally; multiple independent validation, more than two independent samples were adopted; complete accuracy assessment, discrimination was assessed in both training and validation (internal or external) sets.
